# Modeling Red Blood Cell Metabolism in the Omics Era

**DOI:** 10.3390/metabo13111145

**Published:** 2023-11-11

**Authors:** Alicia Key, Zachary Haiman, Bernhard O. Palsson, Angelo D’Alessandro

**Affiliations:** 1 Department of Biochemistry and Molecular Genetics, University of Colorado Denver, Anschutz Medical Campus, Aurora, CO 80045, USA; alicia.m.key@cuanschutz.edu; 2 Department of Bioengineering, University of California, San Diego, CA 92093, USApalsson@eng.ucsd.edu (B.O.P.); 3 Bioinformatics and Systems Biology Program, University of California, San Diego, CA 92093, USA; 4 Department of Pediatrics, University of California, San Diego, CA 92161, USA

**Keywords:** red blood cells, erythrocytes, systems biology, metabolomics, omics, genome-scale metabolic models, personalized medicine, transfusion

## Abstract

Red blood cells (RBCs) are abundant (more than 80% of the total cells in the human body), yet relatively simple, as they lack nuclei and organelles, including mitochondria. Since the earliest days of biochemistry, the accessibility of blood and RBCs made them an ideal matrix for the characterization of metabolism. Because of this, investigations into RBC metabolism are of extreme relevance for research and diagnostic purposes in scientific and clinical endeavors. The relative simplicity of RBCs has made them an eligible model for the development of reconstruction maps of eukaryotic cell metabolism since the early days of systems biology. Computational models hold the potential to deepen knowledge of RBC metabolism, but also and foremost to predict in silico RBC metabolic behaviors in response to environmental stimuli. Here, we review now classic concepts on RBC metabolism, prior work in systems biology of unicellular organisms, and how this work paved the way for the development of reconstruction models of RBC metabolism. Translationally, we discuss how the fields of metabolomics and systems biology have generated evidence to advance our understanding of the RBC storage lesion, a process of decline in storage quality that impacts over a hundred million blood units transfused every year.

## 1. Introduction

Approximately 25 out of 30 trillion cells in the human body are red blood cells (RBCs), which make up for >99% of the corpuscular blood fraction [1]. RBCs carry oxygen through the body, from the respiratory system to peripheral tissues. RBCs play an essential function in the transport and exchange of oxygen and carbon dioxide in circulation, a task that is fulfilled by a single class of extremely abundant proteins, hemoglobins—counting ~250–270 million copies per cell [2]. To this end, RBCs have evolved to maximize oxygen-carrying capacity by removing non-essential cellular components for this task—nuclei and organelles [3]. Of note, the almost complete inability [4] to de novo synthesize proteins in response to environmental stimuli has contributed to the evolutive pressure that favorably selected mechanisms of metabolic regulation of RBC physiology, including modulation of hemoglobin allostery to facilitate oxygen binding at high oxygen saturation in the lung and oxygen release under hypoxic districts, where oxygen demand is high [5]. Therefore, understanding RBC metabolism and its regulation is essential to understanding physiological regulation by oxygen and thus, virtually, almost every aspect of health and disease. In addition, since hemoglobin is a tetramer with binding sites for four oxygen molecules, at full oxygen saturation, each RBC can carry up to 1 billion molecules of oxygen per cell. Since over two thirds of bodily iron is in RBC hemoglobin and oxygen-hemoglobin iron kinetics generate reactive oxygen species, RBCs are equipped with mechanisms of metabolic defense against oxidant stress that are not influenced by the synthesis of new antioxidant enzymes. As such, investigating red blood cell antioxidant metabolism and its regulation in health and disease offers clues on the role of metabolism in health and disease, especially for disease that have oxidant stress as an etiological factor [5].

While the primary purpose of an RBC is to carry oxygen, RBCs contain more than 77 active transporters that can carry metabolites to and from tissues, making them a vehicle for studying diverse physiological processes [3,6]. As such, as they travel from arteries to narrow capillaries in peripheral tissues, RBCs directly or indirectly interact with virtually all organs as a sort of circulating organ system [7]. Therefore, understanding how RBC metabolism is regulated by (and regulates in return) their milieu is of relevance to systems physiology.

Despite the apparent simplicity, proteomics studies of RBCs have determined that the residual <10% of the RBC dry weight beyond hemoglobin is made up of up to 3000 proteins (i.e., 1.5 times as many as a simple unicellular organism like *L. lactis*) [8], with many proteins exhibiting active enzymatic activity as inferred from metabolic flux studies with stable isotope-labeled tracers [9]. These observations contributed to superseding the classic concept of RBCs as a mere bag of hemoglobin, whose biology and—given the lack of mitochondria—metabolism represent a simplified version of that of other cell types, the latter exclusively relying on a handful of key metabolic pathways. In this review, we will discuss how recent advances in proteomics have helped to overcome this concept, while introducing novel concepts like the “exposome”. Indeed, as they travel through the circulatory system, RBCs release and uptake small molecule metabolites from the plasma, including metabolites that are introduced to the bloodstream of an individual through diet, lifestyle, pharmaceutical, environmental, or other exposures: the so-called “exposome” [10]. Further, individual characteristics such as age, sex, exercise, and body mass index also affect RBC physiology.

Understanding RBC metabolism has also significant applications in transfusion medicine. Over 110 million units of packed RBCs are transfused each year into patients worldwide, making blood transfusion the most common iatrogenic intervention after vaccination [3]. During refrigerated storage for up to 42 days, RBCs experience biochemical, morphological, and metabolic changes collectively known as the “storage lesion”, which has deleterious effects on the quality and efficacy of transfused blood products [11].

This review will proceed by discussing relevant RBC metabolic processes, prior work in systems biology in unicellular organisms and RBCs, prior work in deep learning on RBC morphology and metabolic network reconstruction, personalized medicine and systems biology, and potential directions for future work around the area of systems biology of RBCs and personalized transfusion medicine [12].

## 2. Red Blood Cell Metabolism

**Figure 1:**
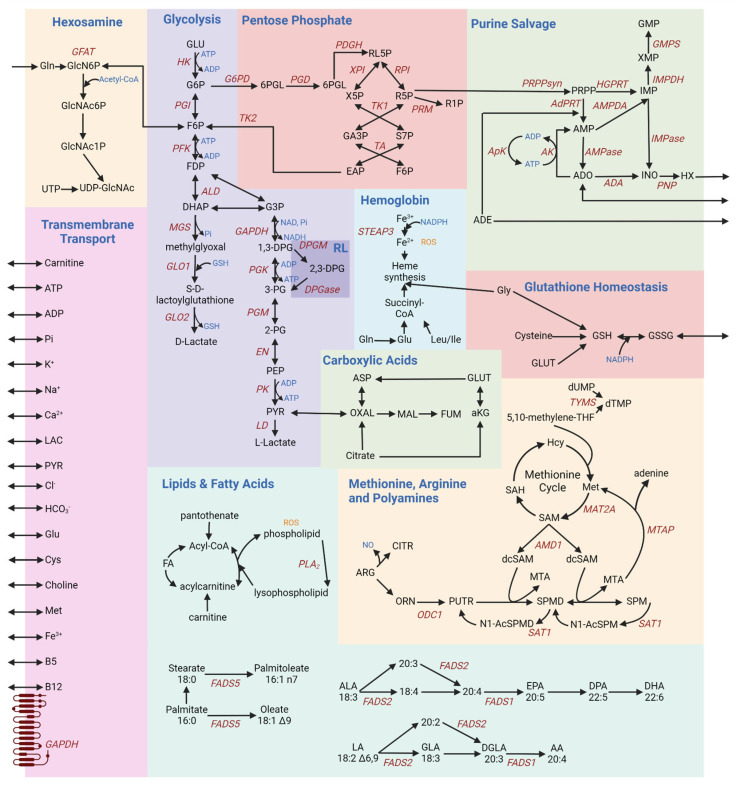
A schematic overview of RBC metabolism [3,9,14,15,16,17,18,19,20,21]. Each box provides a schematic of different metabolic pathways, summarized as follows: glycolysis—the sole energy-generating pathway in mature RBCs, owing to the lack of mitochondria; inhibitory interactions between glycolytic enzymes and the N-terminus of band 3 regulate glycolytic fluxes as a function of hemoglobin oxygenation state; hemoglobin—deoxyhemoglobin modulates glycolysis by outcompeting glycolytic enzymes for the N-term region of band 3; pentose phosphate—NADPH production to fuel all the major antioxidant systems; glutathione homeostasis—regulates the RBC redox potential; purine salvage—synthesis of high-energy purines, and salvage of purine deamination by oxidation and breakdown; hexosamine—production of hexosamine substrates to fuel protein glycosylation, contributing to blood group phenotypes; membrane transport—various ions and small molecule metabolites are transported across the RBC membrane to and from the circulation environment, for example in an ATP- or cation-dependent fashion; methionine, arginine, and polyamines—production of putrescine, spermine, and spermidine; lipids and fatty acids—maintenance of the RBC membrane; carboxylic acids—despite the lack of mitochondria, cytosolic isoforms of Krebs cycle enzyme contribute to the homeostasis of reducing equivalents in the mature RBC.

Ten main metabolic pathways work in concert within a RBC (Figure 1). A collection of payloads are transported into and out of the cell via various mechanisms of energy (adenosine triphosphate—ATP) or ion-facilitated transmembrane transport (Figure 1). In light of the loss of mitochondria during maturation from reticulocytes to mature RBCs, erythrocytes rely solely on glycolysis (Figure 1) for the net generation of two molecules of ATP per oxidation of each glucose molecule, after accounting for the consumption of two molecules of ATP at the hexokinase (EC 2.7.1.1) and phosphofructokinase (EC 2.7.1.56) rate-limiting steps [3]. ATP synthesis occurs at the phosphoglycerate kinase and pyruvate kinase steps. Hemolytic disorders are the most obvious clinical manifestations driving diagnosis of rare genetic mutations in the regions encoding for these enzymes [13]. The end products of glycolysis are pyruvate and lactate, which can both be transported out of the cell via monocarboxylate transporters. Since glycolysis requires NAD+ to sustain the substrate-level phosphorylation reaction catalyzed by glyceraldehyde 3-phosphate dehydrogenase (EC 1.2.1.9), a reaction that concomitantly generates NADH, oxidation of NADH back to NAD+ is achieved in RBCs via two main mechanisms: under low stress conditions, this conversion occurs via lactate dehydrogenase (EC 1.1.1.27) during its catalysis of pyruvate to lactate; when oxidant stress is high, ferric iron reduction upon oxidation of hemoglobin iron is converted back to the ferrous state by methemoglobin reductase, an enzyme that uses NADH as a cofactor, generating NAD+. Pyruvate can also be transaminated to alanine, a reaction that was not observed to occur in mature RBC until recent biochemical and metabolic flux analysis studies with stable isotope-labeled substrates.

The Rapoport–Luebering (RL) shunt is a pathway branching off glycolysis at the triose phosphate step to generate 2,3-diphosphoglycerate (DPG) (Figure 1). By stabilizing the tense deoxygenated state of hemoglobin, DPG allosterically modifies hemoglobin to facilitate oxygen release [22]. As such, DPG levels are elevated in response to hypoxia (e.g., acclimatization to high altitude) [22]. Both PFK (EC 2.7.1.56) and DPG mutase (BPGM, EC 5.4.2.11)—rate-limiting enzymes of glycolysis and the Rapoport–Luebering shunt—have optimal activities at alkaline pH, boosting ATP and DPG synthesis, as well as lactate and protons to favor oxygen off-load from hemoglobin, through a combination of allostery and the so-called Bohr effect. When re-entering glycolysis, DPG contributes to the synthesis of a molecule of ATP, while bypassing one ATP synthesis steps of glycolysis. Like for PK, the frequency of mutations to the gene coding for BPGM is higher in regions where malaria is endemic [23].

The pentose phosphate pathway (Figure 1) produces reduced adenine dinucleotide phosphate (NADPH), an essential cofactor in multiple antioxidant processes [24]: the glutathione system, glutathione peroxidase (EC 1.11.1.9), catalase (EC 1.11.1.6), peroxiredoxins, glutaredoxins, thioredoxin reductase (EC 1.8.4.8) system, biliverdin reductase B7 (EC 1.3.1.24), and the ascorbate–tocopherol axis [24]. The rate-limiting enzyme of this pathway is glucose 6-phosphate dehydrogenase (G6PD [25], EC 1.1.1.49), which is coded by an extremely polymorphic gene in human. As a result, over 500 million people around the world suffer from different degrees of deficient activity of this enzyme, a condition that predisposes RBCs to hemolysis [26] upon oxidant insults and exposures (e.g., sulfa drugs) [25].

Most importantly, NADPH is critical for the conversion of oxidized glutathione to its reduced form (GSSG and GSH, respectively; Figure 1) [27]. Glutathione homeostasis protects against oxidative damage by reactive oxygen species (ROS), such as ROS produced during heme synthesis (Figure 1). Hemoglobin is a sink for intracellular glutathione in an oxygen dependent fashion, through glutathionylation of hemoglobin C93—a residue neighboring the critical histidine residue involved in the coordination of iron within the prosthetic heme group of hemoglobin [28]. Even across mammals [29], species-specific heterogeneity in the number and reactivity of C residues on hemoglobin subunits could thus contribute to intracellular glutathione pools and redox homeostasis, a phenomenon that could be modeled in silico through species-specific reconstruction models of RBC metabolism [30].

Glutathione synthesis is controlled by the availability of cysteine and is fueled by glutamine-derived glutamate. Of note, RBCs can exchange glutamate for cystine, a cysteine disulfide, via Xc transporters that regulate cysteine efflux—a hallmark of ferroptosis [31] in other cell types and of eryptosis in mature RBCs [32]. Glutamate can also fuel transamination reactions, generating alpha-ketoglutarate [33,34]. Carboxylic acids like alpha-ketoglutarate are also generated from citrate via cytosolic isocitrate dehydrogenase 1 (EC 1.1.1.41) (Figure 1). Similarly, aspartate conversion to fumarate upon contribution to purine salvage (Figure 1) reactions can favor the synthesis of malate and oxaloacetate via cytosolic isoforms of malate dehydrogenase 1 (EC 1.1.1.37) [14]. Since this pathway contributes to the homeostasis of reducing equivalents, it has been noted that it responds to environmental hypoxia [15]. The presence of an active phosphoenolpyruvate carboxykinase (EC 4.1.1.32) and malic enzyme 1 (NADPH-generating, EC 1.1.1.40), which can synthesize oxaloacetate and malate from pyruvate—or the moonlighting decarboxylase activity of hemoglobin [35]—has been suggested by flux experiments [36], though as of yet unconfirmed by proteomics studies. In erythroid precursors, glycine levels and the production of succinyl-CoA from branched chain amino acids (isoleucine and leucine) fuel heme synthesis, the central component in the oxygen-carrying capacity of hemoglobin. Amino acid catabolism, including glutamate conversion to proline—via the pyrroline 5-carboxylate intermediate in cells equipped with mitochondria—can connect to arginine catabolism in mature RBCs, at least to the extent that cytosolic steps of this pathway are present and active [37]. Indeed, mature RBCs are loaded with arginase 1 (EC 3.5.3.1) [38]—which synthesizes ornithine—and contain trace amounts of active nitric oxide synthase [39]—which generates the potent vasodilator nitric oxide. From ornithine catabolism, the synthesis of polyamines occurs in cells with mitochondria via the enzyme ornithine decarboxylase (Figure 1). However, in mature RBCs, polyamine levels are likely to be affected by cation amino acid transporters that uptake these metabolites from the environment [40]. Polyamines (putrescine (PUTR), spermidine (SPMD), and spermine (SPM))—which are found throughout the body are indeed also found in red blood cells, where they stabilize the membrane [41] and interfere with divalent cation transport (e.g., calcium [42] and iron [43]). While polyamine synthesis is thought to be reliant on mitochondria-specific steps, an abundance of RBC polyamines has been linked to pathophysiological states (e.g., iron deficiency or irradiation [43]) and ethnicity-specific genetic polymorphisms in the rate-limiting step of spermine metabolism—spermine oxidase (EC 1.5.3.16) [44].

Despite the incapacity to synthesize de novo or very long chain fatty acids, RBCs can scavenge lipids from the media. Fatty acid transporters like CD36 are enriched in erythroid progenitors, though they are reportedly lost during the maturation process [45]. As such, membrane lipid composition is affected by the diet [46] and regulates RBC membrane properties, such as membrane bending rigidity as a function of cholesterol content, fatty acyl chain composition (aliphatic chain length and degree of unsaturation [47]). The lipid and fatty acid pathways play a central role in maintaining the RBC membrane against damage from ROS (Figure 1). Fatty acid desaturases are present and active in mature RBCs, and they participate in NADH homeostasis, especially in response to oxidant stress [16]. Sphingolipid metabolism, especially S1P synthesis in response to hypoxia and transport via Mfsd2b [48], plays a key role in the regulation of metabolic fluxes through glycolysis and the pentose phosphate pathway (Figure 1) [49], a phenomenon that in turn participates in the proper function of high-oxygen-consuming organs like the kidney [50] and brain [51]. It has been proposed that S1P can cooperatively bind to deoxyhemoglobin upon its complexing with DPG, further stabilizing the tense deoxygenated state. While beneficial for acclimatization to hypoxia, this mechanism is actually deleterious in the context of sickle cell disease [52], as it promotes sickle hemoglobin crystallization, driving cardiorenal dysfunction [53,54]. Among other lipid classes, phosphatidylserines participate in RBC removal from the bloodstream upon loss of phospholipid asymmetry, a process that is maintained by ATP-dependent flippases like ATP11C (EC 7.6.2.1) [55,56]. Other phospholipids, like phosphatidylcholines and phosphatidylethanolamines, can contribute methyl group for oxidant stress-induced isoaspartyl protein damage repair via methylation [57] by protein L-isospartyl O-methyltransferase (EC 2.1.1.77) [58]. The main substrates fueling this pathway, methionine and choline, are important antioxidant metabolites in the economy of a mature RBC to combat deamidation and otherwise irreversible alterations in protein backbone orientations in the absence of de novo synthesis capacity [59].

As RBCs circulate through the body, their metabolism responds to the changing levels content and other stimuli they are exposed to. The most common example is the case of hypoxia vs. high oxygen saturation. Under hypoxia, deoxyhemoglobin binds to the N-terminus cytosolic domain of the most abundant RBC membrane protein, band 3—also known as anion exchanger 1, owing to its role in chloride/bicarbonate exchange through which it contributes to the regulation of intracellular pH (Figure 1). In so doing, deoxyhemoglobin displaces glycolytic enzymes from band 3, whereby they are bound and inhibited at high oxygen saturation, when glycolytic fluxes are sacrificed to promote NADPH synthesis via the pentose phosphate pathway and boost antioxidant systems. By promoting release from band 3 of phosphofructokinase (PFK, EC 2.7. 1.11), aldolase (ALD, EC 4.1.2.55) and glyceraldehyde 3-phosphate dehydrogenase (GAPDH, EC 1.2.1.12), deoxyhemoglobin interaction with band 3 favors glucose oxidation via the Embden–Meyerhof–Parnas pathway all the way to triose phosphates, concomitantly favoring DPG synthesis via the Rapoport–Luebering shunt, to further promote oxygen off-loading and deoxyhemoglobin stabilization [60,61,62]. The hexosamine pathway is crucial for the synthesis of glycans that signal the Rh blood group to the immune system [63].

Such elegant mechanisms have only been elucidated over the past few decades, making the modeling of RBC metabolism much more complex than originally thought. The journey that took place from the earliest reconstruction models of RBC metabolism to the most recent ones is detailed below, after a brief background introduction to systems biology.

## 3. Modeling Cell Metabolism through Systems Biology Approaches

Recent advances in omics technologies have facilitated the comprehensive characterization of cell molecules, from genes to transcripts, proteins, and metabolites. Accompanying this characterization is the need to understand the connections among these components. Systems biology studies these connections by finding observable states of these interconnected components [64]. The objective of systems biology is to “generate lists of biological components, determine their interactions, and generate genome-wide data sets” (the interested reader is referred to thematic books on the topic [64,65]). To accomplish this requires four steps. The first step is to define the list of biological components involved in a process. The second step is to reconstruct the interactions between the components and create a “wiring diagram” of the biochemical network. The biochemical network has nodes for the metabolites and links that represent the reaction between the metabolites. The third step is to create in silico models upon a formal mathematical foundation to analyze, interpret, and predict the activity of biochemical networks. The final step is to use the results from the models to generate hypotheses that can be tested experimentally. After all these steps, the in silico models can be improved and refined to make better hypotheses.

Systems biology combines mathematical models of biological systems executed with computational techniques to create models of biological systems executed in silico [66]. Models provided by these studies help elucidate physiology as a function of the relationships between genotype and phenotype. For example, models can find ranges for allowable enzyme activity parameter (k_cat_, K_m_, and v_max_) values in each system. In turn, these parameters help dictate the physiological behavior of a system. However, the concept of “one-model-fits-all” is now being refined in light of the results emerging from high-throughput omics data, pushing systems biology into the arena of personalized medicine. Personalized medicine aims to tailor treatments based on individual patients’ biology. By accounting for interpatient variability, personalized medicine aims to improve the outcomes in complex clinical situations [67]. Through the use of high-fidelity models informed by data collected with high-throughput omics techniques, systems biology holds the potential to impact personalized medicine. Before discussing this perspective, we will briefly summarize a historical perspective of systems biology applications, from unicellular organisms to RBCs and more complex systems.

## 4. Systems Biology in Unicellular Organisms

Systems biology approaches have been previously leveraged to map and analyze metabolic networks in both unicellular organisms and RBCs, demonstrating the versatility of systems biology techniques used to study relatively simple systems [36,68,69,70]. Essential to the relevance of these approaches is the capacity to generate prediction based on varying conditions, predictions that can be empirically tested in the lab. Eventually, iterative feedback between models, predictions, empirical data testing, and model refinement leads to a reconstruction solid enough to hold a reliable predictive capacity, making the need for empirical testing secondary to the identification of the desired starting conditions and final outcomes. In explicit terms, this can, for example, translate to the opportunity to generate in silico models of unicellular fermentative organisms, to identify the substrate levels and limiting reactions that optimize fermentative outcomes, and to then guide the actual testing of such conditions. This example has evident immediate industrial applications, as the examples below will illustrate.

*L. lactis* produces lactic acid from glucose or lactose via the Embden–Meyerhof glycolytic pathway. One finds glucose, phosphoenolpyruvate (PEP), glucose 6-phosphate (G6P), pyruvate, fructose 1,6-bisphosphate (FBP), 3-phosphoglycerate (3-PGA), and lactate on this pathway [66]. *L. lactis* produces lactic acid during periods of glucose abundance and, crucially, can survive periods of glucose absence before restarting lactate production. Voit et al. asked how *L. lactis* does this. They approached this question from two angles. First, using in vivo nuclear magnetic resonance (NMR) spectroscopy of non-growing cells, they monitored the accumulations and depletions of glucose, lactic acid, and intermediates at 30 s intervals after adding a pulse of carbon-13-labeled glucose. Second, they simulated the system with a generalized mass action (GMA) representation of all metabolites along the kinetic pathway. *L. lactis* uses PEP to phosphorylate glucose to G6P. Hence, they reasoned that if the PEP pool was wholly depleted, lactic acid production could not restart when glucose became available again. Consistent with this reasoning, the NMR observations and simulations showed a pool of PEP built up after glucose depletion. G6P could be produced when glucose was re-introduced to the cells. They performed two more tests (one each with the model and NMR) to test whether this PEP pool found in the experiment and simulation would restart glycolysis. First, they added a second pulse of carbon-13 glucose to the NMR experiment 23 min after the first. Second, they added a second pulse of carbon-13 glucose in the simulation. In both cases, they observed that lactate would accumulate again after the re-introduction of glucose to the system. Taken together, the experiments and simulations pointed to a regulatory system managing the pool of PEP.

To explain the accumulation of PEP during periods of glucose starvation, they hypothesized that pyruvate kinase (PK), an enzyme on the pathway, would no longer be activated by FBP and that phosphate accumulation would inhibit PK also. Therefore, PK could no longer turnover PEP to pyruvate. Thus, they reasoned that PEP would accumulate in the pathway. To validate this reasoning, they simulated another model incorporating regulatory feedback (linear feedforward, initially formulated by Savageau) of glucose to pyruvate. The advantage of the simpler model is that it was optimized to study regulation in metabolic networks. When they executed this simulation, they found that PEP would accumulate when the supply of glucose was interrupted. Thus, by using simulation alongside experiments, they tested and validated their hypothesis.

While the *L. lactis* example above is an example of a simple model of a simple biochemical network in a prokaryotic organism, models can be expanded in terms of scale of metabolic reconstruction and the complexity of the organisms they model. In terms of model complexity, commonly used genome-scale metabolic model models (GEMs) are based on knowledge bases incorporating information about genes, reactions, and proteins within organisms, provide a platform for analysis of omics data [71], and enhance the understanding of organism function at a cellular level [72]. GEMs are used to create models of *E.* coli, yeast, and humans [71]. In terms of organism complexity, the next step we will examine in this review is species of yeast such as *S. cerevisae*, which has recently seen the reconstruction of the GEM Yeast8 [71].

Studies of *S. cerevisae* have many applications, ranging from uses in bioengineering (where yeasts are used as a cell factory) to uses as a model organism in biomedical research [71]. The reconstruction of Yeast8 was used to model strain-specific metabolic capabilities, as measured by substrate utilization by 1011 yeast strains. Yeast8 is curated and developed by a community of researchers as open-source software.

## 5. Reconstructions of Human Systems

Significant work has been carried out by many teams to make open-source genome-scale metabolic models (GEMs) of metabolism in humans and other organisms. GEMs provide a framework to study the genotype–phenotype relationship and place the analysis of high-throughput metabolomics data into a larger context [73]. Many of these GEMs are aggregated on the Metabolic Atlas platform. The Metabolic Atlas includes Human-GEM, Yeast-GEM, Fruitfly-GEM, Mouse-GEM, Rat-GEM, Worm-GEM, and Zebrafish-GEM [74]. Aggregating GEMs on an open-source platform, these GEMs have been used in a wide variety of research projects.

As an example of what GEMs include and can accomplish, we will consider the Recon series of models: Recon 1 (published in 2007) [75], Recon 2 (published in 2013) [76], and Recon 3D (published in 2018) [77]. These reconstructions are based on gene sequencing data, literature searches (bibliomics), metabolomics, proteomics, and (in the case of Recon 3D) protein structure data. Further modeling work can be performed based on these reconstructions. Each generation of the reconstruction adds further information upon the earlier version. Unique metabolites modeled started with 1509 in Recon 1, increased to 2626 in Recon 2, and finally increased to 4140 in Recon 3D. Similarly, the genes or open reading frames (ORFs) in the models increased from 1496 in Recon 1 to 1789 in Recon 2, and finally to 3288 in Recon 3. In an improvement over both Recon 1 and Recon 2, Recon 3D added 12,890 three-dimensional protein structures to the reconstruction.

## 6. Systems Biology of RBCs

Classic studies in the field have originally proposed a three-step approach to systems biology in RBCs. First, they suggested untargeted data collection on RBCs using integrated omics techniques. Next, they suggested using computational models from systems biology to generate hypotheses. They suggested testing these hypotheses with targeted omics approaches as the third step. Finally, these steps could be repeated, and the hypotheses successively refined in subsequent iterations [78].

This iterative approach of combining untargeted data collection, computational modeling and analysis, and experimental verification is essential to ensure that computational models produce the correct output and model the system as intended. Downs and others use the simile of a “three-legged stool” to advance knowledge of biochemical pathways with computational models of metabolism. In their model, the stool consists of standard experimental techniques with mass data collection of data with omics techniques and computational analysis. Taken together, all three approaches lead to robust scientific understanding. However, when any one piece is taken out, they argue that the quality of the science will suffer [79]. Similarly, Yurkovich and colleagues propose an iterative workflow of data collection, data analysis, and computational modeling [80]. This review explores how all three approaches can be taken together to advance understanding of RBC metabolism.

Recognizing the need to provide a framework for the analysis of massive datasets from high-throughput omics studies, Bordbar and colleagues developed iAB-RBC-283, a knowledge base of the metabolic network of the RBC [6]. Significantly, iAB-RBC-283 was derived from proteomics data. One of the applications of the iAB-RBC-283 reconstruction is to assist efforts to find metabolic biomarkers in the human RBC [6]. Metabolic biomarkers are metabolites that are potentially useful in disease diagnosis and tracking because they exhibit changes in concentration during disease states. In models, the changes in concentration can be predicted using flux variability analysis (FVA), which finds the space of accessible flux states which satisfy reaction directionality and stoichiometry [81]. One use of FVA is to predict the metabolic signatures of the activity of enzymatic drug targets. Using this iAB-RBC-283, the authors mapped 267 small molecule metabolites, 292 intracellular reactions, 77 transporters, and enzymatic targets of 85 FDA-approved drugs (as of 2011). The team used FVA to predict the metabolic signatures of these enzymatic drug targets. Of note to personalized medicine, the Bordbar team also found 35 morbid single nucleotide polymorphisms related to RBC pathologies. Bordbar et al. studied RBC storage lesion with both experimental metabolomics and a simulation of reaction flux states using Constraint Based Reconstruction and Analysis techniques [82]. They found a decay process exhibiting three metabolic states over the timespan of days 0 to 10, days 10 to 17, and days 17 to 42. Using both experimental and simulation techniques, they found certain pathways changing in magnitude and direction during those metabolic states. The significant pathways they found with this analysis were those including transport and glycolytic enzymes implicated in spherocytosis, anemias, and other hemolytic diseases. Such in silico analysis with iAB-RBC-283 and similar models can establish putative biomarkers and point to interesting paths for experimental analysis.

In the iAB-RBC-283 study, the team found that their knowledge base predicted that erythrocytes contained at least 142 known targets of approved small-molecule medications (these small molecules are known as xenometabolites and, taken together, are part of the exposome). Later, Nemkov and colleagues demonstrated that a xenometabolite identified in that knowledge base, ranitidine affects RBC resting in blood storage units. In this case, Nemkov et al. posited and observed that, if RBCs were supplemented with ranitidine during storage, the quality of the RBCs would be improved through the up-regulation of glycolysis [10].

Yurkovich and colleagues have proposed using omics data generated by investigations of RBC storage lesions as a platform for systems biology modeling and study in a way that contributes to the body of knowledge in transfusion medicine [80]. In an initial study using a systems biology approach, they used principal components analysis (PCA) to find three so-called “metabolic inflection points” that correspond to the depletion of adenine outside the RBCs and the accumulation of hypoxanthine and xanthine in storage. Using this initial data analysis, they created a mechanistic cell-scale model capable of quantitative predictions about 2,3-DPG and citrate [80]. The citrate findings were later experimentally validated [14]. The model’s findings then informed subsequent experimental questions addressed in other studies. First, the three-phase model of metabolic decay was found in different additive solutions [14,36]. Second, it was found that adenine was not the direct cause of the metabolic shifts observed in the different phases [83]. Third, it was found that the RBC metabolic network was robust against changes caused by changes in temperature [84]. Finally, no clear storage advantages were found by supplementing units with alternative sugars such as fructose and mannose [85].

Taken together, these studies show how initial observations from machine learning and data analysis can be used to create a computational model which later can inform further targeted experimental work. However, such models do not capture the heterogeneity of RBC metabolism as a function of genetics and non-genetics factors, an aspect that the introduction of high-throughput omics strategies has shed light on over the last five years.

## 7. High-Throughput Metabolomics and Systems Biology towards Personalized Medicine

According to Redekop and Mldasi, there are three major definitions of personalized medicine [86]. The first definition is using knowledge about a patient to predict treatment response. The second is using knowledge about a patient to predict disease prognosis. The third is to use knowledge about a patient to predict disease susceptibility. In our context, knowledge is derived from a patient’s personal genome, transcriptome, proteome, and metabolome. The prediction would be performed using personalized systems biology models. The prediction would be enabled by linking the patient’s omics profile to what Hartmanshenn et al. referred to as a patient’s clinical phenotype: the traits that are relevant in a clinical treatment setting.

As an example of personalized systems biology, Bordbar et al. created personalized kinetic models of RBCs based on metabolomic data from 24 individuals [87]. Their models used a Mass Action Stoichiometric Simulation (MASS) approach. MASS modeling provides a data-driven and scalable approach to study both the steady-states and dynamics of metabolic network reconstructions [68,69,88]. MASS models are condition-specific models derived from mass action rate laws and multi-omic data to enable computation of pseudo-elementary rate constants (PERCs, single values that represent traditional kinetic parameters of E_0_, K_m_, and k_cat_), thereby circumventing parameterization challenges typically associated with traditional kinetic parameters. K_m_ and k_cat_ depend on the exact protein-coding exon sequence among individuals. Hence individualized changes to the coding sequence potentially change the PERC associated with those values for that protein. Indeed, the Bordbar team found a significant correlation between PERCs and minor allele frequencies (MAFs). In further support of their model’s biological relevance, they found timescales of the dynamics on the order of milliseconds to seconds, which are relevant to fast reactions near equilibrium and key parts of RBC pathways. They found that most inter-individual variation was within dynamics on the circulation time scale. Finally, they studied the effects of the antiviral drug ribavirin in RBC metabolic pathways by adding ribavirin kinetic expressions to the individualized models. Using classification trees based on PERCs as predictors, they separated the individuals into RBV responder and non-responder groups. The classification revealed that PERCs for phosphoglycerate kinase and adenine transport could potentially be important mechanisms in RBV-induced hemolytic anemia.

While this review generally focuses on high throughput omics techniques that generate data for large values of metabolites, another interesting direction is studying a small number of metabolites to predict the future trend of values for a larger number of metabolites. There are two advantages of using a smaller number of metabolites as predictors. First, targeted measurements of a small number of metabolites can yield more detailed insights about the system those metabolites are found in. Second, targeting fewer metabolites makes cost-effective measurement techniques more accessible in a wider variety of settings [89]. In another study by Yurkovich and Yang, they used the metabolomic measurement of only five biomarkers (glucose, hypoxanthine, lactate, malate, and xanthine) to predict the concentration profile of 84 other metabolites with an ensemble of linear output-error models [89].

RBC as a model of all cells can potentially move systems biology into personalized medicine. As a simplified eukaryotic cell, RBCs can serve as a tractable system of study for creating systems biology models. As a cell that travels throughout the body, RBCs can serve as windows onto many systems of clinical relevance [87].

## 8. Future Research Paths

Building on the prior work mentioned here, future research paths can potentially involve (1) connecting donor genotypes to the phenotype of markers of storage lesion and (2) modeling the kinetic response of RBC reactions to hypoxic stress. First, as mentioned above, Bordbar et al. found that pseudo-elementary rate constants (PERCs), which encapsulate E_0_, K_m_, and k_cat_, vary among individuals because of genetic variation that affects the enzymes in question. They suggested a follow-up question necessitating conducting a GWAS study: how does it affect the PERCs [87]? Building on this, another follow-up would be to study metabolic pathways implicated in the RBC storage lesion. How would the genotypes and other properties of individuals within a population of donors affect the pathways in their stored RBCs and, ultimately, the propensity of their blood to hemolyze? Prior work has modeled glucose starvation in *L. lactis* with a generalized mass action (GMA) model [66]. An analog to this glucose starvation seen in RBCs is oxygen deprivation (hypoxia), as experienced by individuals at high altitudes. Similarly, RBCs need to survive and carry scarce oxygen during hypoxia, such as experienced at high altitudes, which was studied in the AltitudeOmics project described previously [22]. Multiple RBC metabolic networks are implicated in this process. Another follow-up would be to model RBC metabolic networks to find ways RBCs may regulate their metabolism in the relative absence of oxygen.
